# Regulatory Functions of *Nilaparvata lugens* GSK-3 in Energy and Chitin Metabolism

**DOI:** 10.3389/fphys.2020.518876

**Published:** 2020-11-25

**Authors:** Yan-Juan Ding, Guo-Yong Li, Cai-Di Xu, Yan Wu, Zhong-Shi Zhou, Shi-Gui Wang, Can Li

**Affiliations:** ^1^Guizhou Provincial Key Laboratory for Rare Animal and Economic Insect of the Mountainous Region, Guizhou Provincial Engineering Research Center for Biological Resources Protection and Efficient Utilization of the Mountainous Region, College of Biology and Environmental Engineering, Guiyang University, Guiyang, China; ^2^College of Life and Environmental Sciences, Hangzhou Normal University, Hangzhou, China

**Keywords:** *Nilaparvata lugens*, RNA interference, glycogen synthase kinase 3, glycogen and trehalose metabolism, chitin metabolism

## Abstract

Glucose metabolism is a biologically important metabolic process. Glycogen synthase kinase (GSK-3) is a key enzyme located in the middle of the sugar metabolism pathway that can regulate the energy metabolism process in the body through insulin signaling. This paper mainly explores the regulatory effect of glycogen synthase kinase on the metabolism of glycogen and trehalose in the brown planthopper (*Nilaparvata lugens*) by RNA interference. In this paper, microinjection of the target double-stranded GSK-3 (ds*GSK-3*) effectively inhibited the expression of target genes in *N. lugens*. *GSK-3* gene silencing can effectively inhibit the expression of target genes (glycogen phosphorylase gene, glycogen synthase gene, trehalose-6-phosphate synthase 1 gene, and trehalose-6-phosphate synthase 2 gene) in *N. lugens* and trehalase activity, thereby reducing glycogen and glucose content, increasing trehalose content, and regulating insect trehalose balance. *GSK-3* can regulate the genes chitin synthase gene and glucose-6-phosphate isomerase gene involved in the chitin biosynthetic pathway of *N. lugens*. *GSK-3* gene silencing can inhibit the synthesis of chitin *N. lugens*, resulting in abnormal phenotypes and increased mortality. These results indicated that a low expression of *GSK*-3 in *N. lugens* can regulate the metabolism of glycogen and trehalose through the insulin signal pathway and energy metabolism pathway, and can regulate the biosynthesis of chitin, which affects molting and wing formation. The relevant research results will help us to more comprehensively explore the molecular mechanism of the regulation of energy and chitin metabolism of insect glycogen synthase kinases in species such as *N. lugens*.

## Introduction

Rice is one of the most important food crops grown in large quantities in Southeast Asian countries (Kang et al., 2019). However, rice is threatened by hundreds of pests throughout many stages, from planting to storage (Ghaffar et al., 2011). *Delphacidae* are one of the most serious rice pests, with the family including insects such as *Laodelphgax striatellus*, *Sogatella furcifera*, and *Nilaparvata lugens*. Since *N. lugens* has a single feeding habit feeding on rice, and is also a carrier of rice rough stunt virus (RRSV) and grass stunt virus (Zhu et al., 2017), it has become the most serious type of *Delphacidae* (Jing et al., 2017; Zhang et al., 2018). At present, most of the chemical pesticides are used to control *N. lugens*, but because of its resistance to a large number of insecticides, the control effect is not satisfactory. In addition, the use of chemical pesticides poses a strong threat to the ecological environment (Diptaningsari et al., 2019).

Glucose metabolism is an important energy metabolism process in living organisms, supplying energy for life activities such as the growth and development of organisms (Carlson et al., 2018). Glycogen is the main form of glucose stored in insects and supplies energy according to the needs of different tissues (Prats et al., 2018). In animals, glucose can be converted to glycogen under the catalysis of glycogen synthase (Klotz and Forchhammer, 2017; Chang et al., 2018), and glycogen degradation requires the action of glycogen phosphorylase (Yamada et al., 2018). Glycogen is mainly stored in the fat bodies of insects and is converted to glucose or trehalose if necessary (Duran et al., 2012; Tang et al., 2012). In insects, glycogen is not a major energy substance, but it is used together with trehalose as a spare sugar to maintain glucose availability (Santiago-Martínez et al., 2016; Prats et al., 2018).

Trehalose is a non-reducing disaccharide unique to insects because it forms the main hemolymph sugar, also known as the “blood sugar” of insects (Zhang et al., 2017b). In fact, trehalose is synthesized by glucose under the catalysis of trehalose-6-phosphate synthase (Matsuda et al., 2015). In addition to providing energy and functioning as a carbon source, trehalose also has bio-protective properties that protects cells and proteins in extreme environments such as cold, oxidation, hypoxia, and dryness (Matsuda et al., 2015; Wen et al., 2016). The degradation of trehalose occurs mainly during the production of glucose under the catalysis of trehalase, which provides energy for life activities such as insect flight. There are two forms of trehalase in insects, soluble trehalase and membrane-bound trehalase (Tang et al., 2017). The synthetic substrates of trehalose, uridine diphosphate glucose and phosphate-6-glucose, can be derived from the decomposition of glycogen (Mitsumasu et al., 2010). Therefore, glycogen metabolism in insects is closely related to the formation and utilization of trehalose.

Trehalase can regulate chitin synthesis in insects (Zhang et al., 2017a). Chitin is the main structural material that constitutes the complex exoskeleton of insects and is found in many insects and arthropods (Zhu et al., 2016). In addition to trehalase, the chitin biosynthetic pathway also includes hexokinase (HK), glucose-6-phosphate isomerase, and glutamine; fructose 6-phosphate transaminase (GFAT), glucosamine-6-phosphate N-acetyltransferase (GNPNA), phosphor acetylglucosamine mutase (PAGM), UDP-N-acetylglucosamine pyrophosphorylase (UAP), and chitin synthase are essential for insect growth and development (Merzendorfer, 2006; Guo et al., 2019). Chitin is the main component of the peritrophic membrane (PM) in the midgut of insects. PM is the natural immune barrier of insects, which can resist the invasion of bacteria, viruses, and other pathogens (Wang and Granados, 2010). Studies on insects such as *Drosophila*, *Bombyx mori*, and *Locust* have found that the obstruction of chitin synthesis can lead to abnormal phenomena such as insect molting, increased mortality, and ovarian hypoplasia (Yang et al., 2015; Xu et al., 2017; Liu and Klein, 2018). Tang et al. (2016) and Chen et al. (2018) found that after RNA interference inhibited the expression of the *N. lugens* trehalase gene and trehalose synthase gene, the chitin content was significantly reduced, and high mortality and difficulty in molting were observed. However, there are few studies on the metabolism of chitin in insects with respect to the Glycogen synthase kinase (*GSK-3*) gene.

In recent years, there have been more and more studies on insect energy metabolism. In addition, various metabolic pathways of insects have become increasingly clear (Arrese and Soulages, 2010; Yang et al., 2014). Glycogen synthase kinase 3 is a key enzyme located in the middle of the sugar metabolism pathway (Mury et al., 2016). Woodgett et al. (1993) isolated and purified two subtypes from skeletal muscle, GSK-3α and GSK-3β, which are encoded by two different genes and are widely expressed in different tissues and cells (Maqbool et al., 2016). *GSK-3* is involved in the regulation of various signaling pathways, such as insulin, Wnt/β-catenin, Hedgehog, and Notch signaling pathways, which play important roles in regulating cell differentiation, metabolism, apoptosis, and gene expression (Wei and Wei, 2015; Khan et al., 2017; Sato and Shibuya, 2018). In insects, *GSK-3* can inactivate glycogen synthase by phosphorylating glycogen synthase, inhibit the final step of glycogen synthesis, and also block the transmission of the insulin signaling pathway to inhibit glycogen synthesis (Frame and Cohen, 2001; Contreras et al., 2016). The conversion between glycogen and trehalose is inseparable, so the expression of *GSK-3* has an important influence on the anabolism of glycogen and trehalose.

The main purpose of this study was to microinject double-stranded RNA into *N. lugens*, thereby silencing the expression of the corresponding gene and achieving RNA interference. This method has been widely used in insect gene research in species such as *N. lugens* (Yu et al., 2013; Qiu et al., 2016; Shi et al., 2016). Once the expression of *GSK-3* in insects is abnormal, the process of glucose metabolism in the body will be unbalanced, and the development of insects, molting, and other life activities will be abnormal (Avonce et al., 2006; Mitsumasu et al., 2010; Shukla et al., 2015). Therefore, this study aimed to investigate the regulation of glycogen synthase kinase on energy metabolism and the chitin synthesis pathway in *N. lugens*. It provides a theoretical basis for pest control through energy and chitin metabolism.

**Graphical Abstract:**
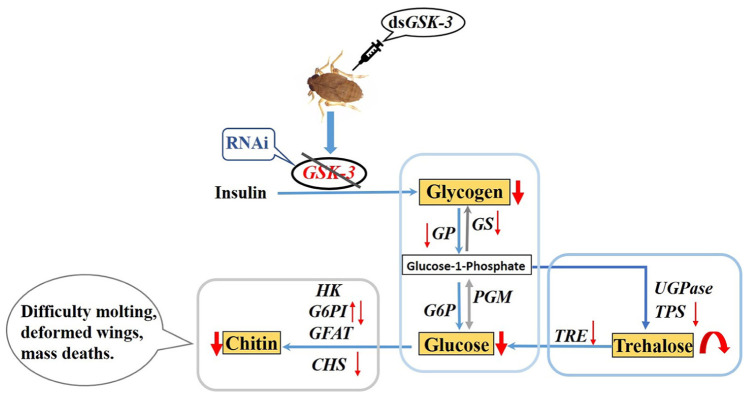
Regulation of *GSK-3* on energy and chitin metabolism of *Nilaparvata lugens*.

## Materials and Methods

### Insects

All the *N. lugens* used in this study were collected from the China Rice Research Institute and were kept in our laboratory. Rice varieties were insect-resistant rice TN1 (Taichung Native 1). Before rice planting, we first immersed rice seeds in warm water at about 70°C for about 10 min to break dormancy; then, seeds were soaked in tap water and placed in a 30°C artificial climate incubator for 24 h. Then, we washed the seeds several times with tap water, wrapped the seeds with wet gauze, and then placed them in a 30°C artificial climate incubator for 24–48 h. Seeds were germinated and sown in plastic pots, and appropriate fertilization promoted seedling growth. After the rice seedlings grew to about 10 cm, they were planted in the field. At the middle of the tillering stage, rice was moved to the insect cage, and rice was replaced every 2−3 days. The conditions for feeding *N. lugens* were as follows: temperature 26°C ± 1°C, photoperiod 16 h/8 h, and relative humidity of 70%.

### Total RNA Extraction and cDNA Synthesis

Total RNA from *N. lugens* was extracted according to the Trizol kit instructions (Invitrogen, Carlsbad, CA, United States). After extraction, the mass of total RNA was detected by 1% agarose gel electrophoresis, and then the concentration and purity of RNA were measured using a NanoDrop 2000 spectrophotometer (Thermo Fisher Scientific, Waltham, MA, United States). The Prime Link^®^ RT Reagent Kit (NARISHIGE, Japan) with gDNA Eraser Kit was used to configure the system and synthesize the first strand of cDNA.

### Synthesis of dsRNA

We designed and synthesized specific primers based on *GSK-3* dsRNA specific fragments. Then, PCR amplification was performed, and the amplified product was subjected to T cloning. The cross-PCR reaction was then carried out using a primer with a T7 promoter. Related primer sequences are shown in Table 1. The synthesis of ds*GSK-3* was performed according to the instructions of the T7 RiboMAX^TM^ Express RNAi System kit (Promega Corporation, Madison, United States), and the concentration of ds*GSK-3* was determined by a NanoDrop^TM^ 2000 spectrophotometer. The same method was used to synthesize *GFP* dsRNA as a control group (Tang et al., 2010; Zhao et al., 2014). In addition, a trehalose solution and a glucose solution at a concentration equal to dsRNA were prepared.

**TABLE 1:** Primers used for the synthesis of dsRNA for *GFP* and *GSK3* genes.

**Gene**	**Application type**	**Primer set**	**Forward primer (5′–3′)**	**Reverse primer (5′–3′)**
Nl*GSK-3*	dsRNA synthesis	dsNl*GSK-3* dsNl*GSK-3*-T7	CTGCGACAGCGGCGAAATG T7-CTGCGACAGCGGCGAAATG	CGGTGACAGATGCCCAGCGAGT T7-CGGTGACAGATGCCCAGCGAGT
*GFP*	dsRNA synthesis	dsNl*GFP* dsNl*GFP*-T7	AAGGGCGAGGAGCTGTTCACCG T7-AAGGGCGAGGAGCTGTTCACCG	CAGCAGGACCATGTGATCGCGC T7-CAGCAGGACCATGTGATCGCGC
T7	dsRNA synthesis		GGATCCTAATACGACTCACTATAGG	

### Microinjection of *N. lugens*

The injection group of this experiment included the ds*GSK-3* only injection group, the ds*GSK-3* and glucose mixed injection group, the ds*GSK-3* and trehalose (Sigma-Aldrich, Saint Louis, MO 63103, United States) mixed injection group, and the ds*GFP* injection group as a control. For the mixed injection group, ds*GSK-3* was mixed in equal volume with an equal concentration of trehalose or glucose solution before injection. The dsRNA was injected into a standard capillary to determine the volume that the microinjector (NARISHIGE, Japan) pumped each time. Then, we adjusted the volume of the pumped dsRNA by nitrogen pressure so that the pumped volume was in accordance with the amount required for injection. Fifth instar *N. lugens* was anesthetized with CO_2_, and then placed in a disposable culture dish with an agar plate on the abdomen. The injection site was the softer part of the first pair of feet in the middle of the foot. The injection volume of each *N. lugens* was 200 ng, with 100 injections per treatment group. The samples were taken at 48 and 72 h after injection for determination of trehalose, glucose, glycogen content, and trehalase activity and the expression of related genes (Tang et al., 2017).

### Expression Studies of Key Genes After *GSK*-3 RNAi

The expressions of key genes in the energy metabolism pathway, insulin signaling pathway, and chitin synthesis pathway were detected by qRT-PCR at 48 and 72 h after injection of *N. lugens*. Parallel sampling was taken; three tubes per treatment group, five *N. lugens* per tube, and three tubes of parallel cDNA were obtained for each sample and were stored in a −80°C refrigerator. During the experiment, three samples of cDNA per tube were obtained, and nine data points were obtained from three tubes of parallel cDNA. Each sample was presented as the average ± standard error to ensure the reliability of the data. The qRT-PCR reaction system (Bio-Rad Laboratories Inc.) (10 μL) was as follows: 5 μL SYBR Premix Ex Taq (SYBR Green Premix Ex Taq, Takara, Japan); 0.4 μL upstream/downstream primer; 1 μL cDNA; and 3.2 μL sterile ultrapure water. Quantitative primers are shown in Table 2, with 18S as the internal control gene. The reaction procedure was as follows: pre-denaturation at 95°C for 10 s, melting at 95°C for 5 s, annealing at 59°C for 30 s, 40 cycles (Zhang et al., 2017b).

**TABLE 2:** Primers used for qRT-PCR measurements of metabolism key genes.

**Gene name**	**Genebank number**	**Forward primer (5′–3′)**	**Reverse primer (5′–3′)**
QNl18S	JN662398.1	CGCTACTACCGATTGAA	GGAAACCTTGTTACGACTT
QNl*GSK-3*	XM_022340458	GGAAAGTTGAATCAAAGTGCTCG	AGGCTTTTGCCAGGGATG
QNl*PGM1*	KU556839.1	TTCTCGGTTGGTGGTGC	CCTTCAGCCTGGGACAT
QNl*PGM2*	KU556840.1	CGTTACAGGCTACGGAAGT	GACCCAAAGCAGTCAAA
QNl*GP*	KU556838.1	GCTGCCTATGGCTATGGTATTC	TCTGAGTGTTGACCCACTTCTTG
QNl*GS*	KU556837.1	GCTCCAAAGCCTATGTTTCTACTG	TGGTAACCCCTGTCCCTCA
QNl*UGPase*	KU556842.1	ATACAAGATGGCGGCTAA	TTGTGGCAGTTGATAGAGC
QNl*TPS1*	GQ397450	AAGACTGAGGCGAATGGT	AAGGTGGAAATGGAATGTG
QNl*TPS2*	KU556826	AGAGTGGACCGCAACAACA	TCAACGCCGAGAATGACTT
QNl*TPS3*	KU556827	GTGATGCGTCGGTGGCTAT	CCGTTCATCATTGGGCATAGT
QNl*TRE1-1*	FJ790319	GCCATTGTGGACAGGGTG	CGGTATGAACGAATAGAGCC
QNl*TRE1-2*	KU556829	GATCGCACGGATGTTTA	AATGGCGTTCAAGTCAA
QNl*TRE2*	GQ397451	TCACGGTTGTCCAAGTCT	TGTTTCGTTTCGGCTGT
QNl*HK*	KU556830	GGTGCGAGAAGAAGTGAAG	GTGAAACCCATTGGTAGAGT
QNl*GFAT*	KU556833	CCTCCCAGTTCATCTCGC	CCAAGTTCTTCAAACCCTTTAT
QNl*G6Pase*	KU556841.1	AGACCCTGGCAGTAGAATAG	GGGAAGTGAGCCGAAAT
QNl*G6PI1*	KU556832.1	GTTCACGGTCGTCTGGAAAG	TGACTGCTCCGTTTCACTCT
QNl*G6PI2*	KU556831.1	AACAAGGCGACATGGAATCG	ACCATTTGTTCCTGGTTCGC
QNl*CHS1*	AEL88648	CCGCAAACGATTCCTACAGA	AGGTCCTTGACGCTCATTCC
QNl*CHS1a*	JQ040014	TGTTCTTGCTACAACTCAATAAA	ACACCAATCCGATAGGCTC
QNl*CHS1b*	JQ040013	GCTGTCTTTGCTTTCTTCAT	ACACCAATCCGATAGGCTC
QNlInR1	KF974333	GAGTGCAACCCGGAGTATGT	TCTTGACGGCACACTTCTTG
QNl*InR2*	KF974334	CTCTTGCCGAACAGCCTTAC	GGGTCGTTTAGTGGGTCTGA
QNl*Ilp1*	KF974340	AACGATGCTGACTTGCAGATT	CGTACACGCGGAATAAATCA
QNl*Ilp2*	KF974341	TTCTCAGCCGCTCTAGCAAT	CAGACGAAGGATCAGGGAA G
QNl*Ilp3*	KF974342	ATACTGCGGCCAATAGCAAG	TCTCAATCCCCAAAATCAGC
QNl*Ilp4*	KF974343	TCCCGGACAGTTCTCACTTT	TTGTATTCTCCGGAGGCAAG

### Determination of Trehalose, Glucose, Total Glycogen Content, and Trehalase Activity

Each treatment and control material was added, after which 100 μL of phosphate buffered saline (PBS) was added, ground, and then added to 100 μL of PBS; sonication was performed until to no blocky structure was observed, after which the samples were crushed, added to 800 μL of PBS, and centrifuged at 1,000 *g* for 20 min at 4°C. Then, 350 μL of supernatant was collected and ultracentrifuged for 60 min at 4°C, 20,800 *g*. The 1,000 *g* centrifugation supernatant was used for determination of the protein concentration, glycogen concentration, and trehalose concentration. The supernatant after ultracentrifugation was used for the determination of the glucose content (supernatant), soluble trehalase activity (TRE1), and protein (Pr1). In addition, the pellet was suspended in PBS and used for the determination of the glucose content (suspension), membrane-bound trehalase (TRE2), and protein (Pr2). The specific steps are described in the kit instructions. In short, 75 μL of 40 mM trehalose (Sigma-Aldrich, Saint Louis, Mo 63103 United States) and 165 μL PBS (pH 7.0) were added into 60 μL 1,000 *g* supernatant. The mixture was immersed in water for 60 min at 37°C and 5 min at 100°C. TRE activity was measured with glucose detection kit (Sigma-Aldrich) using supernatant (50 μL). The protein content in 1,000 *g* supernatant, ultracentrifugation supernatant, and suspension was determined by BCA Protein Assay Kit (Beyotime, China). Trehalose content was measured by anthrone method, and 30 μL 1% H_2_SO_4_ was added to 30 μL samples. The mixture was bathed in water at 90°C for 10 min and in ice for 3 min. After 30 μL 30% KOH was added, the mixture was again bathed in water at 90°C for 10 min and in ice for 3 min. Then, 600 μL developer (0.02 g fluorenone + 100 ml 80% H_2_SO_4_) was added, water bathed at 90°C for 10 min, and cooled in an ice bath. The absorbance of the sample was measured at 630 nm with a microplate reader. One hundred sixty microliter of the supernatant was obtained after centrifugation of 1,000 *g* for the determination of glycogen content. Six hundred microliter of anthrone sulfate reagent was added to the sample, placed in a water bath at 90°C for 10 min, and then cooled in an ice bath. The absorbance of the sample was measured at 625 nm with a microplate reader. The Glucose Assay Kit (SIGMA) was used to determine the glucose content in the supernatant and pellet suspension obtained after centrifugation at 20,800 *g*. 150 μL of the sample was placed into an EP (Eppendorf), and then 30 μL of 2N H_2_SO_4_ was added to stop the reaction after 30 min in a 37°C water bath, and the absorbance of the sample at 540 nm was then measured using a microplate reader.

### Chitin Analysis

The detection method of the proportion of chitin in the brown planthopper refers to the “Insect Biochemical and Molecular Biology Experimental Technology” compiled by Cao and Gao (2009), with appropriate modifications. Two glass tubes were connected to both ends of a rubber tube, about 30–50 cm long. Another test tube with a rubber plug with holes was taken and the glass tube was inserted at one end of the rubber tube into the rubber plug, and the other end was passed into the water to prevent the lye from splashing out. 40 *N. lugens* was taken, three groups were paralleled, and then dried in an oven at 50°C. The *N. lugens* dried to a constant weight was weighed and recorded as W1. The dried insect body was poured into a test tube, 5 mL saturated potassium hydroxide solution was added, and it was heated in a glycerin bath at 160°C until the insect body developed a transparent film. The residue was filtered, rinsed carefully with water, dried in an oven at 50°C, weighed, and counted as W2. The relative content of chitin (%) = (W2 / W1) × 1.26 × 100 (%), where 1.26 is the ratio of relative molecular mass of acetylglucosamine to glucosamine.

### Statistical Analyses

The average CT value of the three replicate wells was used for calculation, and the final data were the mean ± standard error. Then, the 2^–ΔΔCT^ formula was used to calculate the CT value of the ds*GFP* group injected into *N. lugens*. The 2^–ΔΔCT^ calculation formula is as follows:

$$2^{-\Delta \,\Delta \,\text{CT}}=2^{\begin{array}{c} -[\left(\mathrm{CT}\mathrm{of}\mathrm{experimental}\mathrm{group}-\mathrm{CT}\mathrm{of}\mathrm{experiment}18S\right)\\ -\left(\mathrm{CT}\mathrm{of}\mathrm{control}\mathrm{group}-\mathrm{CT}\mathrm{of}\mathrm{control}18S\right)] \end{array}}.$$

Charts were drawn using Excel software, statistical analysis was performed using STATISTICA 8.0 and SigmaPlot 10.0, and the significance difference test was performed using one-way ANOVA (^∗^ indicates *P* < 0.05, the difference is significant; ^∗∗^ indicates *P* < 0.01, the difference is extremely significant).

## Results

### Expression of *GSK*-3 After RNAi

**FIGURE 1:**
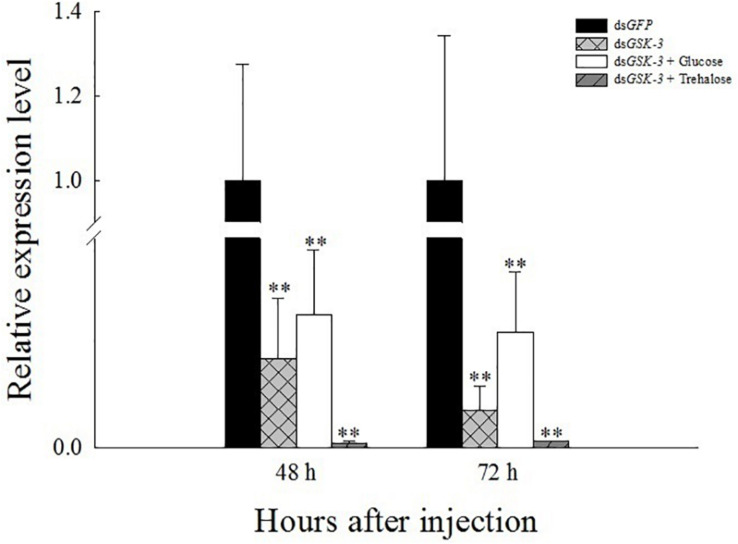
Relative expression level of *N. lugens GSK-3* 48 and 72 h after RNA interference. ^∗∗^Indicates significant differences at *P* < 0.01. Error bars is the standard error.

Compared with the injection of the ds*GFP* group, the expression levels of *GSK-3* mRNA in the three treatment groups were significantly decreased at 48 and 72 h after injection. Among them, the ds*GSK-3* and trehalose mixed injection group decreased most obviously (*P* < 0.01) (Figure 1). This result indicates that the injection of ds*GSK-3*, a mixture of ds*GSK-3* and glucose, a mixture of ds*GSK-3*, and trehalose can significantly reduce the expression of *GSK-3*.

### Effects of *GSK*-3 RNAi on Glycogen, Glucose, and Trehalose Contents

**FIGURE 2:**
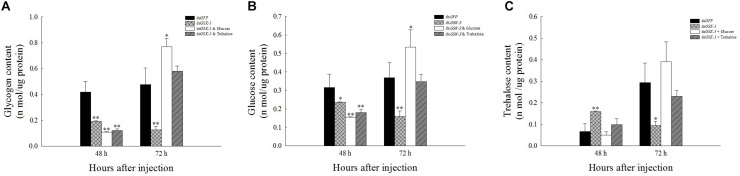
Effect of *GSK-3* RNA interference on glycogen, glucose, and trehalase contents of *N. lugens.* Changes in glycogen **(A)**, glucose **(B)**, and trehalose **(C)** contents in *N. lugens* were observed at 48 and 72 h after injection in each treatment group. ^∗^Indicates significant differences at *P* < 0.05 and ^∗∗^indicates significant differences at *P* < 0.01. Error bars is the standard error.

Compared with the injection of the ds*GFP* group, glycogen and glucose levels were extremely significantly decreased or significantly decreased 48 h after injection (Figures 2A,B), and the trehalose content increased extremely significantly 48 h after ds*GSK-3* injection (*P* < 0.01) (Figure 2C). The glycogen, glucose, and trehalose contents were significantly decreased or extremely significantly decreased 72 h after ds*GSK-3* injection (Figure 2); the glucose and glycogen contents were significantly increased after injection of a mixture of ds*GSK-3* and glucose (*P* < 0.05) (Figures 2A,B), and the glycogen and glucose contents were restored to a level similar to that observed in the control group after injection of a mixture of ds*GSK-3* and trehalose (Figures 2A,B). In the two mixed injection groups, the change in the trehalose content was not significant (Figure 2C).

### Effects of *GSK*-3 RNAi on Two Kinds of Trehalase in *N.lugens*

**FIGURE 3:**
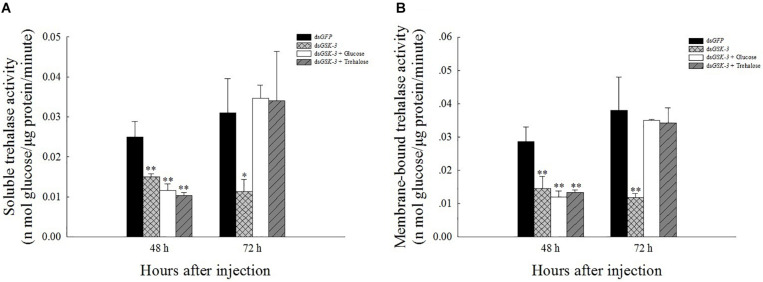
Effect of *GSK-3* RNA interference on trehalase activity of *N. lugens.* The soluble trehalase **(A)** and membrane-bound trehalase **(B)** activities of *N. lugens* were observed 48 h and 72 h after injection in each treatment group. ^∗^Indicates significant differences at *P* < 0.05 and ^∗∗^indicates significant differences at *P* < 0.01. Error bars is the standard error.

The results of the trehalase activity assay showed that the soluble trehalase and membrane-bound trehalase activities of the three treatment groups were extremely significantly decreased 48 h after injection (*P* < 0.01) (Figures 3A,B). The two trehalase activities were significantly reduced 72 h after ds*GSK-3* injection (*P* < 0.01) (Figures 3B,C), and the trehalase activity of the two mixed injection groups returned to a level similar to the control group (Figure 3).

### Expression of Chitin Synthesis Pathway-Related Genes After *GSK*-3 RNAi

**FIGURE 4:**
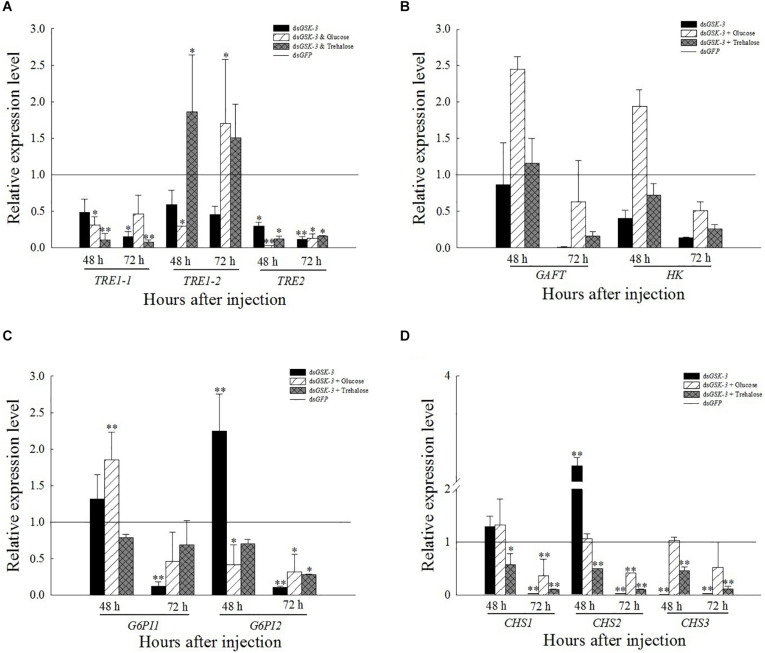
Expression levels of regulated genes in the chitin synthesis pathway after *GSK-3* RNA interference. The relative expression levels of three *TRE* genes **(A)**, *GFAT* and *HK* genes **(B)**, *G6PI* genes **(C)**, and *CHS* **(D)** genes after dsRNA injection compared with the control ds*GFP*. ^∗^Indicates significant differences at *P* < 0.05 and ^∗∗^indicates significant differences at *P* < 0.01. Error bars is the standard error.

The expression of *TRE1*-1 and *TRE2* decreased or decreased significantly compared with the injection of ds*GFP* after RNAi inhibited the expression of *N. lugens GSK*-3. The expression of *TRE1*-2 increased significantly 48 h after injection of a mixture of ds*GSK-3* and trehalose (*P* < 0.01) and decreased slightly 72 h after injection, and the expression of *TRE1*-2 also increased significantly after injection of a mixture of ds*GSK-3* and glucose (*P* < 0.01, Figure 4A). There was no significant change in the expression of *GFAT* and *HK* genes after ds*GSK-3* and a mixture of ds*GSK-3* and glucose injection (Figure 4B). After inhibition, the expression level of *G6PI* showed a trend of increasing first and then decreasing (Figure 4C). The expression of *CHS1a* was extremely significantly increased 48 h after ds*GSK-3* injection (*P* < 0.01), the expression of *CHS1b* was extremely significantly decreased after injection (*P* < 0.01), and the expression of *CHS* was not significantly changed 48 h after injection of a mixture of ds*GSK-3* and glucose. Injection of a mixture of ds*GSK-3* and trehalose showed significant inhibition of *CHS* expression (*P* < 0.05). The expression levels of *CHS* in each treatment group decreased or decreased extremely significantly 72 h after injection (Figure 4D).

### Expression of Genes Involved in the Energy Metabolism Pathway After *GSK-3* RNAi

**FIGURE 5:**
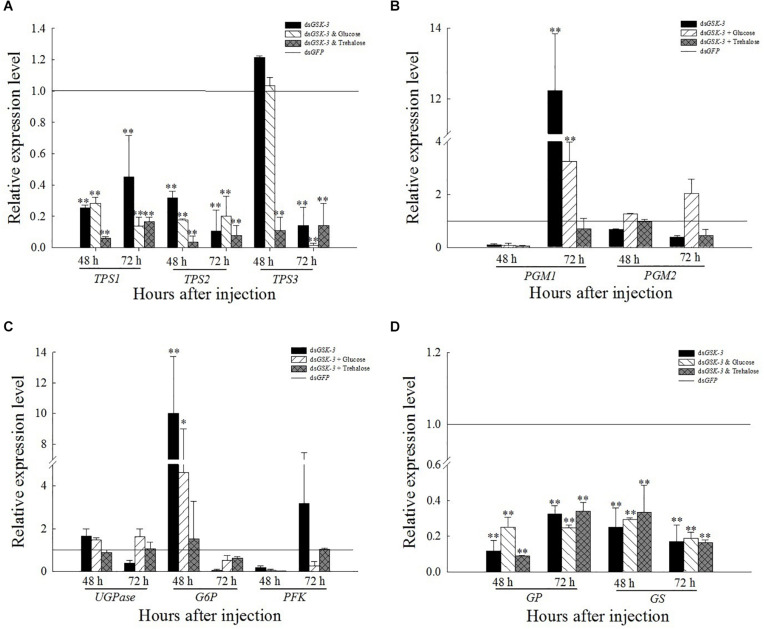
Expression levels of regulated genes in the energy metabolism pathway after *GSK-3* RNA interference. The relative expression levels of *TPS* genes **(A)**, *PGM* genes **(B)**, *UGPase*, *G6P*, and *PFK* genes **(C)**, and *GP* and *GS* genes **(D)** were compared with the control ds*GFP* after injection in each treatment group. ^∗^Indicates significant differences at *P* < 0.05 and ^∗∗^indicates significant differences at *P* < 0.01. Error bars is the standard error.

Compared with the control group ds*GFP*, the expression levels of *TPS1* and *TPS2* were extremely significantly decreased after treatment. The expression of *TPS3* increased but was not significant 48 h after injection of ds*GSK-3* and a mixture of ds*GSK-3* and glucose (Figure 5A). The expression of *PGM1* was extremely significantly increased 72 h after ds*GSK-3* and a mixture of ds*GSK-3* and glucose injection, and there was no significant change in *PGM2* expression (Figure 5B). The expression of *GS* and *GP* decreased extremely significantly after injection in each treatment group (Figure 5D). The expression of *G6P* increased significantly 48 h after ds*GSK-3* injection and decreased after 72 h. The expression of *UGPase* and *PFK* did not change significantly (Figure 5B).

### Expression of Insulin Signaling Pathway-Related Genes After *GSK-3* RNAi

**FIGURE 6:**
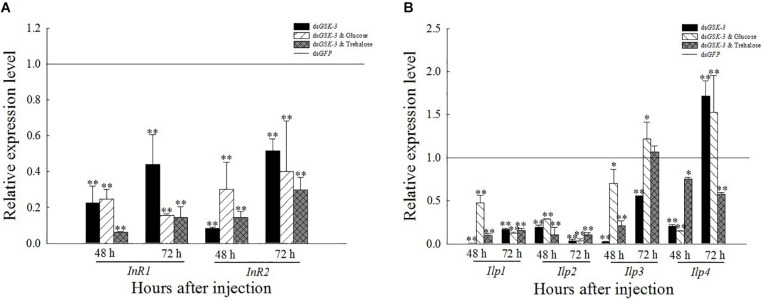
Expression levels of regulated genes in the insulin signaling pathway after GSK-3 RNA interference. The relative expression levels of *InR* genes **(A)** and *Ilp* genes **(B)** were compared with the control dsGFP after injection in each treatment group. ^∗^Indicates significant differences at *P* < 0.05 and ^∗∗^indicates significant differences at *P* < 0.01. Error bars is the standard error.

After RNAi inhibited the expression of *GSK*-3, the expression levels of *InR*, *Ilp1*, and *Ilp2* were extremely significantly lower than those of ds*GFP* (*P* < 0.01) (Figure 6). The expression of *Ilp3* and *Ilp4* was significantly or extremely significantly decreased 48 h after injection, and increased extremely significantly 72 h after the injection of a mixture of ds*GSK-3* and glucose. The expression of *Ilp4* increased extremely significantly 72 h after ds*GSK-3* injection (Figure 6B).

### Analysis of Phenotype, Malformation Rate, Mortality, and Chitin Content After *GSK-3* RNAi

**FIGURE 7:**
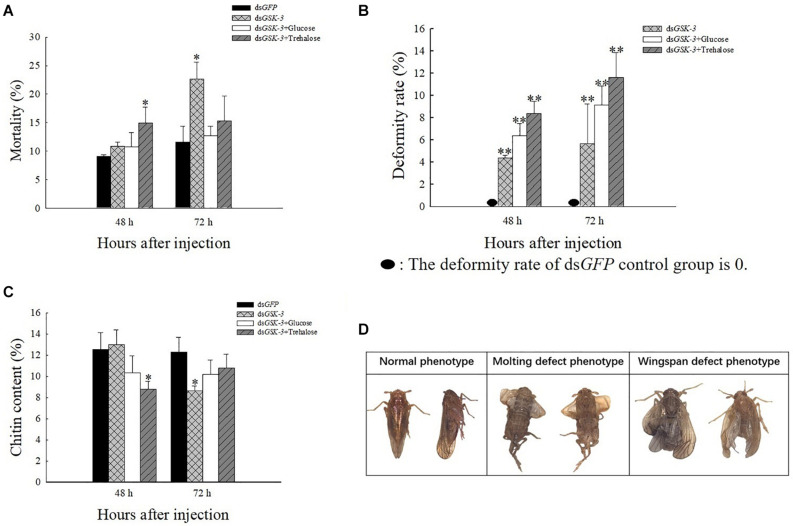
The effect of *GSK-3* gene silencing on the chitin content and phenotype of *N. lugens*. Mortality rate **(A)**, deformity rate **(B)**, chitin content **(C)**, and Normal and abnormal phenotypes of *N. lugens* **(D)** were compared with the control dsGFP after injection in each treatment group. ^∗^Indicates significant differences at *P* < 0.05 and ^∗∗^indicates significant differences at *P* < 0.01. Error bars is the standard error.

After injection of dsRNA, the death rate of *N. lugens* increased, and various abnormal phenotypes appeared. Compared with the control group injected with ds*GFP*, the mortality rate was significantly increased at 48 h after mixed injection of ds*GSK-3* and trehalose and 72 h after the interference of ds*GSK-3* (*P* < 0.05) (Figure 7A). *N. lugens* in each treatment group showed abnormal phenotypes, including molting deformities and wing deformities (Figure 7D); the deformity rate of the mixed injection group was higher than that of the ds*GSK-3* alone injection group (Figure 7B).

After RNAi treatment, the relative content of chitin in *N. lugens* changed. 48 h after the injection, except for the ds*GSK-3* and trehalose mixed injection group, there was a significant decrease (*P* < 0.05), and the other injection groups had no significant changes. 72 h after ds*GSK-3* injection, the content of chitin in *N. lugens* significantly decreased (*P* < 0.05), while the other injection groups did not decrease significantly (Figure 7C). These results indicate that *GSK-3* gene silencing affects the metabolism of chitin and the formation of wings in *N. lugens*.

## Discussion

RNA interference has great potential for studying insect gene function, mainly by microinjecting dsRNA or siRNA into insects to inhibit gene expression (Xi et al., 2015; Li et al., 2017) or synthesized *in vitro* and topically applied to crops (Murphy et al., 2016). In this study, the expression levels of the *GSK-3* gene were extremely significantly decreased after injection in each treatment group of *N. lugens*, indicating that the RNA interference effect is obvious, and the interference effect can last up to 72 h (Figure 1). Previous studies have found that *GSK*-3 is involved in a variety of signaling pathways and plays an important regulatory role in cells (Liu et al., 2018). *GSK-3* acts on the Ser332 site of insulin receptor substrate-1 (IRS-1) to block the insulin-signaling pathway, thereby regulating glycogen synthesis (Liberman and Eldar-Finkelman, 2005). After insulin enters the insect, it activates *Ilp* and *InR* and then activates phosphatidylinositol kinase (PI3K). Activated PI3K catalyzes phosphorylation of phosphatidylinositol, which in turn causes phosphorylation of the effector Akt, which prevents phosphorylation of glycogen synthase (GSK-3), thereby promoting glycogen synthesis (Woodgett et al., 1993; Nagy et al., 2018). The effect of *Ilp* on glucose metabolism cannot only promote glycogen synthesis but also inhibit gluconeogenesis and conversion of excess sugar into lipids in specific tissues (Semaniuk et al., 2018; Kawabe et al., 2019). The results of this study showed that the expressions of insulin signaling pathway-related genes *InR*, *Ilp1*, *Ilp2*, and *Ilp3* were extremely significantly decreased 48 and 72 h after ds*GSK-3* injection (Figure 6). Only the expression of *Ilp4* increased extremely significantly 72 h after injection (Figure 6B), presumably related to its regulation of the reproduction of *N. lugens* (Lu et al., 2018). At the same time, the glycogen and glucose contents of *N. lugens* were significantly or extremely significantly decreased (Figures 2A,B), indicating that *N. lugens GSK*-3 can regulate glycogen and glucose synthesis through the insulin signaling pathway (McManus et al., 2005).

Trehalose can act as an energy reserve for the life activities of insects under stress, such as starvation and dryness (Yasugi et al., 2017). Under various stresses, trehalose accumulation occurs in insects (Fountain et al., 2016; Tamang et al., 2017; Paithankar et al., 2018). This study found that the trehalose content increased extremely significantly 48 h after ds*GSK-3* injection (Figure 2C), and the two trehalase activities decreased extremely significantly (Figures 3A,B), showing consistency. However, the trehalose content decreased extremely significantly (Figure 2C) and trehalase activity remained significantly decreased 72 h after injection (Figures 3A,B). TPS can affect the energy supply, growth and development, stress recovery, chitin synthesis, and other biological processes of *N. lugens* (Tang et al., 2018). When the expression of *TPS* and *TRE* in the trehalose metabolism pathway of the *N. lugens* was inhibited, the glycogen content decreased (Zhang et al., 2017b). In this study, the expressions of *TPS1*, *TPS2*, and *TRE* were down-regulated after *GSK*-3 interference (Figures 4A, 5A), that is, the accumulation of trehalose in *N. lugens* led to an increase in its content 48 h after GSK-3 RAN interference, but due to the resistance of trehalose synthesis, the content of trehalose decreased 72 h after injection. The expression of *TPS3* increased 48 h after *GSK-3* interference, probably because the structure of *TPS3* is different from that of *TPS1* and *TPS2*, so their functions are also different. Al Baki MA et al. found that the chyme content of *Alpaki* was increased after *Ilp* and *InR* RNAi (Al Baki et al., 2019), which is consistent with the results of this study (Figures 6A,B), that is, glycogen and glucose are converted into trehalose after ds*GSK-3* injection, which leads to an increase in glucose metabolism and maintains the balance of glucose metabolism in insects, which is consistent with previous studies (Mitsumasu et al., 2010; Tang et al., 2012).

The metabolism of glycogen requires a combination of enzymes, mainly regulated by glycogen synthase (GS) and glycogen phosphorylase (GP) (Zhang et al., 2017b). *GSK-3* can phosphorylate glycogen synthase to inhibit glycogen synthesis (Contreras et al., 2016). In our study, the expression of *GS* and *GP* was extremely significantly decreased 48 and 72 h after ds*GSK-3* injection (Figure 5D). In animals, glycogen plays a role in different tissues, and hepatic glycogen can be decomposed into glucose during hypoglycemia to control blood sugar stability; glycogen can also supply energy to organisms through the glycolysis pathway (Roach et al., 2012; Falkowska et al., 2015). In this study, the expression of the *PGM1* gene in the energy metabolism pathway decreased slightly 48 h after injection but increased extremely significantly 72 h after injection (Figure 5B). In addition, the expression of *G6P* associated with glycogen degradation returned to a level similar to that of the ds*GFP* group after a significant increase after injection (Figure 5C; Klotz and Forchhammer, 2017). That is, the expression of *GSK*-3 can inhibit the metabolism of carbohydrates.

The reasonable intake of dietary sugar is essential for the growth and development of insects, while the trehalose of insects is mostly synthesized by the intake of glucose (Thompson et al., 2003; Mattila and Hietakangas, 2017). Under a high-sugar diet, the glucose content in *Drosophila* increased, while the expression of adiponectin (*Akh*) was down-regulated, and the glycogen content was reduced (Gáliková et al., 2015; Hemphill et al., 2018). Our results showed that the glycogen and glucose levels of two mixed injection groups showed extremely significant decreases compared with the control group 48 h after injection; the glycogen and glucose levels increased significantly 72 h after injection of a mixture of ds*GSK-3* and glucose, and the mixed injection group of ds*GSK-3* and trehalose returned to a level similar to the control group (Figures 2A,B). In contrast, the two mixed injection groups had no significant effect on the trehalose content (Figure 2C), but both types of trehalase activity decreased significantly 48 h after injection and rose to a level similar to the control group 72 h after injection (Figures 3A,B). *TRE1* mainly exists in the midgut of *N. lugens* and regulates the decomposition of endogenous trehalose; *TRE2* mainly absorbs and assimilates exogenous trehalose (Tang et al., 2016). Therefore, the expression level of *TRE1*-2 was significantly increased 48 h after a mixture of ds*GSK-3* and glucose injection and 72 h after a mixture of ds*GSK-3* and trehalose injection (Figure 4A). *HK* catalyzes the conversion of glucose to glucose-6-phosphate, and *GFAT* decomposes fructose-6-phosphate. The expression of *GFAT*, *HK*, and *G6PI1* in the chitin pathway increased or significantly increased 48 h after injection of a mixture of ds*GSK-3* and glucose (Figures 4B,C). *In vitro* injection of glucose or trehalose can compensate for the interference of ds*GSK-3* in the energy metabolism and chitin synthesis of *N. lugens*, but it seems that *N. lugens* is more sensitive to changes in the glucose content (Masumura et al., 2000; Ugrankar et al., 2015).

The growth and development of insects are closely related to the biosynthesis of chitin (Merzendorfer and Zimoch, 2003; Muthukrishnan et al., 2018). Trehalose is the first gene in the chitin biosynthesis pathway of insects, which regulates chitin synthase (Zhao et al., 2016; Tang et al., 2017). Previous studies have shown that the inhibition of *CHS* gene expression will lead to a decrease in the content of chitin, abnormal phenotypes and an increase in mortality of *N. lugens* (Yang et al., 2017; Li et al., 2017; Pan et al., 2019). Our results showed a decrease or a significant decrease in *TRE* expression after ds*GSK-3* injection (Figure 4A). At the same time, compared with the ds*GFP* group, the expression of *CHS1* was similar 48 h after ds*GSK-3* injection; the expression of *CHS1a* was extremely significantly increased, the expression of *CHS1b* was extremely significantly decreased, and the expression of three *CHS* genes was significantly decreased 72 h after injection (Figure 4D). At the same time, the content of chitin was significantly reduced 48 h after ds*GSK-3* and trehalose injection and 72 h after ds*GSK-3* injection (Figure 7C). This suggests that ds*GSK-3* has different regulatory effects on different chitin synthase genes, but ultimately inhibits their expression, which may be related to the functional specificity of chitin synthase (Arakane et al., 2009; Merzendorfer, 2011). Our research also found that *N. lugens* had difficulty molting, deformed wings, and increased mortality after ds*GSK-3* injection (Figures 7A,B,D). In addition, the expression of *G6PI* also increased 48 h after ds*GSK-3* injection and decreased after 72 h (Figure 4C). The interference of *GSK*-3 has an effect on the expression of genes involved in the chitin biosynthesis pathway of *N. lugens*.

## Conclusion

*GSK*-3 RNAi can effectively inhibit the expression of target genes in *N. lugens*; *GSK-3* can down-regulate the expression of energy metabolism pathway-related genes and trehalase activity, thereby reducing the glycogen and glucose content, increasing the trehalose content, and regulating insect trehalose balance; *GSK-3* can regulate genes involved in the chitin biosynthesis pathway of *N. lugens*, affecting its chitin synthesis, leading to phenotypic abnormalities and even death.

## Data Availability Statement

All datasets generated for this study are included in the article/supplementary material.

## Author Contributions

Y-JD, G-YL, and CL conceived and manuscript structure design. C-DX, YW, and S-GW performed the current articles collection and related metabolic genes’ analysis. Y-JD, Z-SZ, and CL wrote the manuscript. All authors contributed to the article and approved the submitted version.

## Conflict of Interest

The authors declare that the research was conducted in the absence of any commercial or financial relationships that could be construed as a potential conflict of interest.
